# Effect of Air Pollutants Particulate Matter PM_2.5_, PM_10_, Carbon Monoxide (CO), Nitrogen Dioxide (NO_2_), Sulfur Dioxide (SO_2_), and Ozone (O_3_) on Fractional Exhaled Nitric Oxide (FeNO)

**DOI:** 10.12669/pjms.40.8.9630

**Published:** 2024-09

**Authors:** Sultan Ayoub Meo, Mustafa A. Salih, Joud Mohammed Alkhalifah, Abdulaziz Hassan Alsomali, Abdullah Abdulrahman Almushawah

**Affiliations:** 1 Sultan Ayoub Meo Department of Physiology, College of Medicine, King Saud University, Riyadh, Saudi Arabia; 2 Mustafa Abdalla Mohamed Salih Department of Paediatrics (Neurology Unit), College of Medicine, King Saud University, Riyadh, Saudi Arabia; 3 Joud Mohammed Alkhalifah College of Medicine, King Saud University, Riyadh, Saudi Arabia; 4 Abdulaziz Hassan Alsomali College of Medicine, King Saud University, Riyadh, Saudi Arabia; 5 Abdullah Abdulrahman Almushawah College of Medicine, King Saud University, Riyadh, Saudi Arabia

**Keywords:** Environmental Pollution, Fractional Exhaled Nitric Oxide, FeNO, Airway inflammation, Schools

## Abstract

**Objectives:**

This study aimed to investigate the effect of Environmental Pollutants Particulate Matter PM_2.5_, PM_10_, Carbon Monoxide (CO), Nitrogen Dioxide (NO_2_), Sulfur Dioxide (SO_2_), and Ozone (O_3_) on lung airway inflammation by assessing the Fractional Exhaled Nitric Oxide (FeNO) in students studying in schools located in or away from air-polluted areas.

**Methods:**

This matched case-control cross-sectional study was conducted in the Department of Physiology, College of Medicine, King Saud University, Riyadh, Saudi Arabia from August 2022 to July 2023. In this study, two schools were selected, one was located near a traffic-polluted area (School #1), and the second was located away from the traffic-polluted area (School #2). A total of 300 students were recruited, 150 (75 male and 75 female) students from the school located in a traffic-polluted area, and 150 students (75 male and 75 female) from the school located away from a traffic-polluted area. Environmental pollutants PM_2.5_, PM_10_, CO, NO_2_, O_3_, and SO_2_, were recorded. The Fractional Exhaled Nitric Oxide (FeNO) was measured using a Niox Mino.

**Results:**

The mean concentration of PM_2.5_, PM_10_, CO, NO_2_, O_3_, and SO_2_ were 35.00±0.65 significantly higher in a school located in motor vehicle polluted area compared to a school located away from a motor vehicle-polluted area (29.95±0.32) (p=0.001). The mean values for FeNO were significantly higher (18.75±0.90) among students studying in a school located in the motor vehicle-polluted area compared to students studying in a school located away from the motor vehicle-polluted area (11.26±0.56) (p=0.001).

**Conclusions:**

Environmental pollution can cause lung inflammation among students in schools located in traffic-polluted areas.

## INTRODUCTION

Over the past three decades, environmental pollution has grown to be a global public health threat. The rapid rise in industrialization and urbanization contaminates the entire ecosystem. The air, water, and soil are all impacted by the various sources of pollution, which also change the environment’s composition.^1^ Environmental pollution and the diseases associated with it are caused by type, nature, size, concentration, and duration of exposure to airborne pollutants in the breathing zone.^2^

The biological composition and severity of air pollution can vary based on the geographical location, weather conditions, population, and human activities in each area. Environment and human health are interlinked, and efforts to improve them represent key aspects of sustainable development within the spheres of environment, economy, and society.^3^-^5^

The World Health Organization (WHO) has declared that environmental pollution is a silent killer, causing about seven million people deaths each year, or 15.5 people per minute. Moreover, about 92% of people do not breathe safe air. Pollution affects both human health and global economies as 400 billion US Dollars are spent on subsidizing fossil fuel use.^6^ Environmental pollution is a leading risk factor for several illnesses mainly respiratory^7^, coronary artery diseases^8^, endocrine^9^, nervous system disorders^7^, and cancer.^10^

An increase in motor vehicle fleet size has increased environmental pollution. Evidence suggests that motor vehicles generate enormous quantities of carbon dioxide, carbon monoxide, nitrogen oxide, hydrocarbons, and particulate matter PM_2.5_, and PM_10_, which contaminate the environment and cause environmental pollution.^7^ In many countries, schools are located near busy traffic roads or traffic-related polluted areas. Exposure of children and adolescents to these environmental pollutants, particularly during their physiological developmental age period increases the risk of health-associated issues.^5^ This study aimed to investigate the effect of Environmental Pollution Particulate Matter PM_2.5_, PM_10_, Nitrogen Dioxide (NO_2_), Sulfur Dioxide (SO_2_), and Ozone (O_3_) on The Fractional Exhaled Nitric Oxide (FeNO) in students studying in schools located in air-polluted areas.

## METHODS

This matched case-control “cross-sectional study was conducted in the Department of Physiology, College of Medicine, King Saud University, Riyadh, Saudi Arabia from August 2022-July 2023”.

### Selection of Schools:

In this study, two different schools located in two separate areas were selected. The first school was situated near a motor vehicle-polluted area, within 200 m of the main traffic road. This school was considered a motor vehicle pollutant-exposed school (exposed group, school #1). The second school was located away from motor vehicle-polluted areas, at a minimum of 1,500 m away from the main traffic road. This school was considered a school less exposed to motor vehicle pollutants (control group, school #2).

### Selection of Students:

The students in both schools were recruited based on their voluntary involvement and healthy status, matched by age, height, weight, gender, nationality, and regional, socioeconomic, and cultural background. For example, a student who attends school #1 was matched to a student at school #2.

### Exclusion Criteria:

The known cases of diabetes mellitus, obesity, asthma, COPD, pulmonary tuberculosis or malignancy, cigarette, and shisha smokers, and previous history of exposure to any factory that produces dust or fumes were excluded.^8^ Students whose family members, such as their father or mother were cigarette smokers were also excluded from the study to minimize the passive smoking effect on the lungs.

### Measurements of Air Pollutants:

The airborne particles were determined through integrated sampling systems, and air pollutants were recorded every hour over 24 hours. PM_2.5_, PM_10_, CO, NO_2_, SO_2_, and O_3_ were measured using the MP101M (2.5), MP101M (10), CO12e, AC32e, AF22e, O342e, Air Quality Monitors. The air pollutants data were obtained from the National Center for Environmental Compliance (NCEC), Riyadh, Saudi Arabia. Moreover, daily, air pollutants were recorded from the Air Quality Index (AQI).^11^ All these methods and sources were used to obtain the air pollutant data.

### Measurement of Fractional Exhaled Nitric Oxide (FeNO):

FeNO was measured by using a Niox Mino. The test was conducted in a sitting position, the subject exhaled appropriately. At each session, three correctly performed exhalations were recorded^7^. Subjects were advised to avoid eating, drinking, and/or exercising for at least one hour before the test procedure. Exhaled Nitric Oxide measurements were expressed in ppb.^7^

### Statistical Analysis:

The data were analyzed by using the “Statistical Package for Social Sciences (SPSS for Windows, version 21.0; IBM, Armonk, NY, USA).” The descriptive statistics were presented as means and standard deviations (Mean±SD); an unpaired Student’s t-test (parametric test) was applied to evaluate the difference in the means between the variables. The p-value < 0.05 was considered significant.

### Ethical Statement:

This study was approved by the “Institutional Review Board, College of Medicine, King Saud University, Riyadh, Saudi Arabia” (Ref #21/01099/IRB).

## RESULTS

In this study, two schools were selected, one in a traffic-polluted area (School #1), and the second in an area away from the traffic-polluted area (School #2). The air pollutants include particulate matter PM_2.5_, PM_10_, carbon monoxide (CO), nitrogen dioxide (NO_2_), ozone O_3_, sulfur dioxide (SO_2_), and total pollutants were recorded (Table-I). The mean concentration of environmental pollutants PM _2.5_ in school #1 was 110.64 ± 33.61 significantly higher compared to school #2 (108.86 ± 25.04) (p=0.004). Similarly, PM_10_ in school #1 (60.22 ± 46.04) was significantly higher compared to school #2 (27.38 ± 18.62) (p=0.001).

**Table I:** Environmental pollutant levels in school #1 located in a motor vehicle polluted area and school #2 located away from the motor vehicle polluted area.

Pollutants (24 hs/average)	School # 1	School # 2	Significance level
Particulate Matter PM_2.5_ μg/m3	110.64 ± 33.61	108.86± 25.04	0.004
Particulate Matter PM_10_ μg/m3	60.22± 46.04	27.38±18.62	0.001
Carbon Monoxide (CO) ppm	7.32 ± 3.20	6.65±4.15	0.394
Nitrogen dioxide (NO_2_) DU	11.23±10.76	6.39±3.52	0.001
Ozone O_3_ (DU)	18.11±6.84	28.09±16.65	0.001
Sulfur dioxide (SO_2_) DU	2.36±1.38	2.33±1.15	0.548
Total air pollutants	35.00±0.65	29.95±0.32	0.001

School #1: Located in a traffic-polluted area; School #2: located away from the traffic-polluted area.

The concentration of NO_2_ was 11.32±10.76, significantly higher in school #1 compared the school #2 (6.39 ± 3.52) (p=0.001). Moreover, CO 7.32 ± 3.20 and Sulfur dioxide (SO_2_) 2.36 ± 1.38 in school #1 was high compared to school #2 CO 6.62±4.15 and SO_2_ 2.33±1.15 but did not significantly high. The overall concentration of environmental pollutants PM_2.5_, PM_10_, CO, NO_2_, O_3_, and SO_2_ was 35.00±0.65 significantly higher in school #1 compared to school #2 (29.95±0.32) (p=0.001) (Table-I).

### Demographic variables of the students:

In this study, a total of 300 students were recruited, 150 (75 male and 75 female) students from school #1 located in a traffic-polluted area, and 150 students (75 male and 75 female) from school #2 located away from a traffic-polluted area. The students were selected based on age, gender, health status, height, weight, BMI, ethnicity, and homogenous educational and socioeconomic status. The demographic variables of the students belonging to School #1 and School #2 are sown in Table-II. The overall mean age of students was 13.53±1.20 years. The average age of students from school #1 was (n=150, mean=13.56; SD=1.27), while the mean age of students from school #2 was (n=150, mean=13.50; SD=1.17).

**Table II:** The age, height, weight, and BMI of students (n=300).

Variable	School (n=150 in each school)	Mean	SD	Minimum	Maximum	Significance level
Age (years)	School #1 students	13.56	1.27	11.0	17.0	NS
	School #2 students	13.50	1.17	11.0	16.0	
Height (cm)	School #1 students	161.32	9.62	143.0	183.0	NS
	School #2 students	162.09	9.17	143.50	182.0	
Weight (kg)	School #1 students	62.50	21.33	29.0	140.0	NS
	School #2 students	62.91	17.71	31.0	130.0	
BMI (kg/m)^2^	School #1 students	23.79	6.72	12.00	46.40	NS
	School #2 students	23.91	6.39	12.90	46.12	

NS= non-significant.

The average height of students at school #1 (n=150, mean =161.32; SD=9.62), and school #2 was (n=150, mean =162.09; SD=9.17). While the mean weight of students at school #1 was (n=150, mean=62.50; SD=121.33; and school #2 was (n=150, mean=62.91; SD=17.71). Moreover, the BMI of students at school #1 was (mean=23.79; SD=6.72 and school #2 was (mean=23.91; SD=6.39. The age, height, weight, and BMI matching of the students in both schools was non-significant. It shows that the students in both schools were very well-matched for age, gender, height, weight, and BMI (Table II).

### Fractional Exhaled Nitic Oxide (FeNO):

The comparison of FeNO between students studying in school #1 compared to students studying in school #2 is shown in Table-III. FeNo was significantly increased (18.75±0.90) among the students at school #1 compared to the students studying in school #2 (16.40±0.88) (p=0.002). It was also found that FeNo was significantly increased (15.69±0.52) among the girl students in school #1 compared to the girl’s students studying in school #2 (11.26±0.56) (p=0.001) (Table-III). FeNO was significantly increased in students studying in school #1 located in the traffic-polluted as compared to those studying in school #2 which was located away from the traffic-polluted area. However, there was no significant difference in FeNO levels in boys’ students (Table-III).

**Table-III:** Fractional Exhaled Nitic Oxide (FeNO) levels in students studying in school #1 compared to the students studying in school #2 (n=300).

Parameters	School #1	School #2	Significance level
***Boys Students (n= 150): 75 students in each group***			
FeNO	21.81±1.70	21.53±1.46	NS
***Girl students (n=150): 75 students in each group***			
FeNO	15.69±0.52	11.26±0.56	0.001
***Total Students: 300 (150 in each school)***			
FeNO	18.75±0.90	16.40±0.88	0.02

School #1: Located in a traffic-polluted area; School #2: located away from a traffic-polluted area.

## DISCUSSION

Environmental pollution is the most significant global problem, it affects the climate conditions and has a profound influence on human health.^12^ The present study results revealed that the overall mean values for PM_2.5_, PM_10_, CO, NO_2_, O_3_, and SO_2_ were significantly higher in a school located in a motor vehicle-polluted area. These air pollutants caused increased FeNO levels among students who study in schools located in motor vehicle-polluted areas. FeNO is an important marker of endogenous inflammation, it is a simple and noninvasive test used to examine the inflammatory changes in the lungs. FeNO levels in conjunction with clinical history, and laboratory markers aid in reaching a better diagnosis of respiratory diseases.^13^,^14^

Eckel et al.^15^ assessed the effect of Traffic-related air pollution (TRAP) exposure on airway inflammation by measuring the FeNO among school children. TRAP exposure was associated with elevated levels of FeNO. Adamkiewicz et al.^16^ found that air pollutant PM_2.5_ was associated with an increase in FeNO. Furthermore, Meo et al.^7^ conducted a pilot study and found that FeNO was slightly increased in the students who were exposed to motor vehicle pollution. In another study, Godri et al.^17^ found that exposures to PM_2.5_, traffic and industrial-derived emissions were associated with an enhanced FeNO level in asthmatic children.

Similarly, in the present study, it was identified that the mean values for FeNO were significantly higher among students studying in a school located in the motor vehicle-polluted area compared to students studying in a school located away from the motor vehicle-polluted area. Environmental pollutants cause airway inflammation therefore, FeNo was increased among the students studying in a school located in an air-polluted area. This study offers a new insight into the effects induced by exposure to air pollutants associated with underlying mechanisms in airway inflammation.

### Study strengths and limitations:

This study investigated the impact of environmental pollution on FeNO among school adolescents who are studying in schools located near and away from air-polluted areas. In this study, students were matched for age, height, weight, and BMI levels to minimize the confounding factors. There is a rare such matching in the studies. The first limitation is that this cross-sectional study limits finding the cause-and-effect relationships. The second limitation is that due to the COVID-19 pandemic and lockdown public activities were restricted there was a reduction in air pollution because of decreased industrial, and economic activities, and reduced traffic emissions. It may minimize the actual impact of air pollutants on FeNO.

## CONCLUSIONS

Environmental pollution can cause lung inflammation among students studying in the school located in motor vehicle-polluted areas. To combat this issue, it is crucial to implement effective mitigation strategies, as well as promote sustainable and environmentally friendly transportation options.

## Data Availability Statement:

None.

### Authors Contributions:

**SAM:** Study design, literature review, data collection, analysis, writing and editing the manuscript, He is also responsible for the integrity and accuracy of the manuscript.

**MAS:** Data checking, Review and verification,

**JAK, AHS, AAA:** Literature review, data collection and analysis.
